# Identification of long non‐coding RNA MVIH as a prognostic marker and therapeutic target in acute myeloid leukemia

**DOI:** 10.1002/jcla.23113

**Published:** 2019-11-13

**Authors:** Zhiyong Jiang, Qinghong Yu, Xinguo Luo

**Affiliations:** ^1^ Department of Hematology Jinhua People's Hospital Zhejiang China; ^2^ Department of Hematology The First Affiliated Hospital Zhejiang University Hangzhou China

**Keywords:** acute myeloid leukemia, cell apoptosis, long non‐coding RNA microvascular invasion in hepatocellular carcinoma, risk stratification, survival

## Abstract

**Background:**

This study aimed to investigate the correlation of long non‐coding RNA microvascular invasion in hepatocellular carcinoma (lncRNA MVIH) with disease risk, disease conditions, and prognosis of acute myeloid leukemia (AML), and also to investigate the influence of lncRNA MVIH on AML cell activities in vitro.

**Methods:**

A total of 212 AML patients and 70 controls were consecutively recruited. Their bone marrow mononuclear cells (BMMCs) were isolated, and lncRNA MVIH was detected by reverse transcription quantitative‐polymerase chain reaction. In AML patients, complete remission (CR), event‐free survival (EFS), and overall survival (OS) were assessed. In vitro, lncRNA MVIH expression in AML cell lines was determined, and the effect of lncRNA MVIH on AML cell proliferation and apoptosis was assessed.

**Results:**

LncRNA MVIH expression was increased in AML patients compared to controls, and receiver operating characteristic curve showed that lncRNA MVIH predicted elevated AML risk (area under curve: 0.742 [95% CI: 0.674‐0.810]). In AML patients, no correlation of lncRNA MVIH expression with French‐American‐British classification was observed, while lncRNA MVIH high expression correlated with worse risk stratification. Moreover, lncRNA MVIH expression negatively correlated with CR achievement, EFS and OS. In vitro, lncRNA MVIH was overexpressed in AML cell lines (KG‐1, ME‐1, and HT‐93) compared to normal BMMCs. Furthermore, lncRNA MVIH downregulation reduced KG‐1 cell proliferation but increased apoptosis, whereas lncRNA MVIH upregulation raised HL‐60 cell proliferation but decreased apoptosis.

**Conclusion:**

LncRNA MVIH may not only serve as a prognostic marker but also act as a therapeutic target in AML.

## 1 INTRODUCTION

Acute myeloid leukemia (AML), one of the most common leukemias, is characterized by abnormal proliferation of undifferentiated and non‐functional leukemic blasts in the bone marrow (BM), which results in around 20% of all hematologic malignancy‐related deaths.^1^, ^2^ The current progresses in AML mainly include improvements on treating opportunistic infectious diseases, advances on supportive care, and reduce of complications after allogeneic BM transplantation; however, the therapeutic effects are unsatisfactory, with a 5‐year survival rate of 40% for younger patients (18‐60 years) and a 5‐year survival rate of 10% for the elder patients (age above 60 years), and AML is still a life‐threatening disease.^1^, ^3^, ^4^ Thus, rational development of novel strategies to improve the treatment outcomes of AML is important. Since emerging studies have revealed that the association of molecular aberrations with the prognosis may contribute to guide therapeutic decisions, exploring novel biomarkers that being identified for AML prognosis is desperately needed.^5^, ^6^

Long non‐coding RNAs (lncRNAs) are key members of the non‐coding RNA family that possess limited protein‐coding capacity.^7^ In the post‐genomic era, lncRNAs have been rapidly gaining recognition for their crucial roles across diverse biological processes.^8^, ^9^ Among them, lncRNA microvascular invasion in hepatocellular carcinoma (MVIH), located on *RPS24* gene and encoding a member of the S24E ribosomal proteins, has been reported to correlate with deteriorative disease progression and predict poor survival profiles in several solid tumors, such as hepatocellular carcinoma (HCC), breast cancer, and non‐small cell lung cancer (NSCLC).^10^, ^11^, ^12^, ^13^, ^14^ Moreover, some experiments have disclosed the ability of lncRNA MVIH to promote cell proliferation and repress apoptosis in several tumors.^10^, ^11^, ^14^ All these data emphasized the oncogenic role of lncRNA MVIH in specific solid tumors; however, no evidence about the role of lncRNA MVIH in any hematological malignancies was found, including AML. Considering AML had some common biological characters with solid tumors, for instance, the abnormally exuberant proliferation of malignant cells, we inferred that lncRNA MVIH, a lncRNA promoted cell proliferation in some solid tumors, might also facilitate malignant cell proliferation and contribute to disease initiation in AML. Therefore, to validate this speculation, this study investigated the predictive value of lncRNA MVIH for disease risk and the correlation of lncRNA MVIH expression with some crucial disease conditions as well as prognosis in AML; moreover, we also explored the influence of lncRNA MVIH on AML cell activities in vitro.

## 2 MATERIALS AND METHODS

### 2.1 Subjects

This study consecutively recruited 212 de novo AML patients between January 2015 and December 2018. Patients were eligible for recruitment if they had (a) a diagnosis of de novo AML confirmed by examinations of morphology, immunophenotyping, cytogenetics, molecular cytogenetics, and genetics, (b) age ≥ 18 years; (c) no history of other malignancies, (iv) able to be followed up regularly. While patients were excluded from the study if they were (a) diagnosed as acute promyelocytic leukemia (APL), (b) secondary or relapsed AML, (c) previously treated by radiotherapy, chemotherapy or stem cell transplantation before recruitment, (d) pregnant or breastfeeding women. Furthermore, the current study also enrolled a total of 70 controls including BM donors as well as subjects who underwent BM biopsy for non‐hematological malignant disease.

### 2.2 Ethics statement

This study was approved by Institutional Review Board of Jinhua People's Hospital and carried out in accordance with the Declaration of Helsinki. All enrolled subjects provided the written informed consents before entry to the study.

### 2.3 Sample collection

Bone marrow samples of AML patients were collected before they received treatment. The BM samples of controls were collected when their eligibility was confirmed. After sample collection, the BM mononuclear cells (BMMCs) were isolated by the density gradient centrifugation. Subsequently, the lncRNA MVIH expression in the BMMCs was determined by the reverse transcription quantitative‐polymerase chain reaction (RT‐qPCR).

### 2.4 Induction therapy and response assessment

The induction therapy decisions for AML were based on age, history of prior myelodysplasia or cytotoxic therapy, and performance status. Standard induction regimens were administered to all AML patients, and the regimen generally consisted of 3 days of an anthracycline (eg, daunorubicin 45‐60 mg/m^2^ or an alternative anthracycline at equivalent dose), and 7 days of cytarabine (100‐200 mg/m^2^ continuous iv). Dose reduction was considered for individual patients and decided by their treating physician according to the National Comprehensive Cancer Network (NCCN) Clinical Practice Guidelines in Oncology of AML (version 1.2014). In addition, response assessment was commonly performed between day 21 and 28 after start of induction therapy. Complete remission (CR) was defined as BM with at least 20% cellularity and BM blasts below 5% at steady state after treatment, without cytological evidence of leukemia, no transfusion requirement, leukocyte count above 1*10^9^/L and platelet count above 100*10^9^/L.^15^

### 2.5 Data collection

Baseline clinical data of AML patients were recorded after initial evaluation was completed, and the main clinical data included age, gender, French‐American‐British (FAB) classification, cytogenetics, molecular genetics, risk stratification, and white blood cell (WBC) count. The risk stratification evaluation was based on cytogenetics and molecular genetics, according to the NCCN Guidelines (version 1.2014). Remission status of patients was documented after response assessment. Besides, survival data were collected by conventional follow‐up, which was performed through regular re‐examination, clinic visit or telephone calls. All patients were consecutively followed up to December 31, 2018, resulting in a median follow‐up duration of 19.0 months. Event‐free survival (EFS) was defined as the time interval from initiation of treatment to disease recurrence, progression, or death. Overall survival (OS) was defined as the time interval from initiation of treatment to death. For the patients not known to have disease recurred, progressed, or died at last follow‐up date, they were censored on the data of last visit or on the date of last known to be alive.

### 2.6 Cell culture

Human AML cell lines including KG‐1, ME‐1, HT‐93, and HL‐60 were purchased from Leibniz Institute DSMZ‐German Collection of Microorganisms and Cell Cultures (Braunschweig, German). The cell lines were maintained in 90% Roswell Park Memorial Institute (RPMI) 1640 Medium (Gibco) supplemented with 10% fetal bovine serum (Gibco). Cultures were maintained in a humidified incubator at 37°C in 5% CO_2_. The lncRNA MVIH expression in the cell lines was determined by the RT‐qPCR, and the normal BMMCs isolated from healthy BM donors were used as control.

### 2.7 Transfections

The pRNAT‐U6.1/Neo vectors and the pCMVp‐NEO‐BAN vectors were purchased from BioVector NTCC Inc and used to structure the knock‐down (KD) plasmids and the overexpression (OE) plasmids, respectively. With the use of Lipofectamine 2000 (Invitrogen), the lncRNA MVIH KD plasmids and the negative control (NC) KD plasmids were transfected into KG‐1 cells which were then identified as KD‐MVIH cells and KD‐NC cells, accordingly; and lncRNA MVIH OE plasmids and the NC OE plasmids were transfected into HL‐60 cells that were then marked as OE‐MVIH cells and OE‐NC cells, correspondingly.

### 2.8 Determinations after transfection

At 24 hours after transfection, the RT‐qPCR was carried out in all transfected cells to detect the expression of lncRNA MVIH; and Annexin V/Propidium Iodide (AV/PI) assay with Annexin V‐FITC Apoptosis Detection Kit (R&D) was carried out to evaluate cell apoptosis. Besides, Cell Counting Kit‐8 (CCK‐8; Sigma) was used to measure cell viability in all transfected cells at 0 hour, 24 hours, 48 hours, and 72 hours. Both the CCK‐8 and the AV/PI assay were performed in accordance with the kit manufacturer's instructions.

### 2.9 RT‐qPCR

Total RNA was extracted by RNeasy Protect Mini Kit (Qiagen), and reverse transcription to cDNA was performed by PrimeScript™ RT reagent Kit (Takara). Then, qPCR was conducted using TB Green™ Fast qPCR Mix (Takara), and the results were calculated by 2^−ΔΔ^ *^C^* ^t^ method. Besides, glyceraldehyde‐3‐phosphate dehydrogenase (GAPDH) was applied as the internal reference. Sequences of primers used in RT‐qPCR assay were as follows:

Lnc‐MVIH: Forward: AATTTTGCACATCTGAACAGCC; Reverse: TTCAAAATCCCACTACGCCCA.

GAPDH, Forward: TGACCACAGTCCATGCCATCAC; Reverse: GCCTGCTTCACCACCTTCTTGA.

### 2.10 Statistical analysis

Data analyses and graph plotting were performed by using SPSS 22.0 (IBM) and GraphPad Prism 7.01 (GraphPad Software Inc). Data were displayed as mean value ± standard deviation (SD), median, and interquartile range (IQR) or number (percentage). Comparison of lncRNA MVIH relative expression between AML patients and controls or between CR patients and non‐CR patients was determined by Wilcoxon rank sum test. Comparison of lncRNA MVIH expression among M1, M2, M4, M5, and M6 or among better risk, intermediate risk, and poor risk was determined by Kruskal‐Wallis H rank sum test. Comparisons of lncRNA MVIH high‐expression and low‐expression percentages between CR patients and non‐CR patients or among M1, M2, M4, M5, and M6 or among better risk, intermediate risk, and poor risk were determined by chi‐squared test. Comparison of lncRNA MVIH relative expression among different cell lines was determined by Dunnett's *t* test. Comparison of lncRNA MVIH relative expression between KD‐NC and KD‐MVIH or between OE‐NC and OE‐MVIH was determined by unpaired *t* test. Comparisons of cell proliferation and apoptosis between KD‐NC and KD‐MVIH or between OE‐NC and OE‐MVIH were determined by unpaired *t* test. The performance of lncRNA MVIH in differentiating AML patients from controls was assessed by receiver operating characteristic (ROC) curves and the area under the ROC curve (AUC); also, the sensitivity and specificity at the best cutoff point were calculated. The EFS and OS were illustrated by Kaplan‐Meier (K‐M) curves, and the differences of EFS and OS between lncRNA MVIH high group and lncRNA MVIH low group were determined by log‐rank test. *P* value < .05 was considered as significant.

## 3 RESULTS

### 3.1 Clinical characteristics of AML patients

A total of 212 AML patients (including 107 (50.5%) females and 105 (49.5) males) with mean age of 50.8 ± 15.7 years were enrolled in this study (Table 1). The median WBC value was 15.5 (7.7‐25.9) × 10^9^/L. For FAB classification, 1 (0.5%), 89 (42.0%), 48 (22.6%), 61 (28.8%), and 13 (6.1%) patients presented with M1, M2, M4, M5, and M6, respectively. As for cytogenetics, 101 (47.6%), 21 (9.9%), 19 (9.0%), 17 (8.0%), 7 (3.3%), 4 (1.9%), 4 (1.9%), 4 (1.9%), 3 (1.4%), 3 (1.4%), 1 (0.5%), and 28 (13.2%) patients presented with NK, CK, t(8;21), inv(16) or t(16;16), 11q23, +8, −7 or 7q‐, −5 or 5q‐, t(9;11), t(9;22), t(6;9) and other cytogenetics, respectively. Besides, there were 17 (8.0%) patients with MK, 43 (20.3%) patients with FLT3‐ITD mutation, 23 (10.8%) patients with isolated biallelic CEBPA mutation and 66 (31.1%) patients with NPMI, respectively. Regarding risk stratification, 61 (28.8%), 81 (38.2%), and 70 (33.0%) patients showed better, intermediate, and poor risks, respectively.

**Table 1:** Clinical characteristics of AML patients

Items	AML patients (N = 212)
Age (y), mean ± SD	50.8 ± 15.7
Gender, No. (%)	
Female	107 (50.5)
Male	105 (49.5)
WBC (×10^9^/L), median (IQR)	15.5 (7.7‐25.9)
FAB classification, No. (%)	
M1	1 (0.5)
M2	89 (42.0)
M4	48 (22.6)
M5	61 (28.8)
M6	13 (6.1)
Cytogenetics, No. (%)	
NK	101 (47.6)
CK	21 (9.9)
*t*(8;21)	19 (9.0)
inv(16) or t(16;16)	17 (8.0)
11q23	7 (3.3)
+8	4 (1.9)
‐7 or 7q‐	4 (1.9)
‐5 or 5q‐	4 (1.9)
*t*(9;11)	3 (1.4)
*t*(9;22)	3 (1.4)
*t*(6;9)	1 (0.5)
Others (not included in better or poor risk)	28 (13.2)
MK, No. (%)	17 (8.0)
FLT3‐ITD mutation, No. (%)	43 (20.3)
Isolated biallelic CEBPA mutation, No. (%)	23 (10.8)
NPMI, No. (%)	66 (31.1)
Risk stratification, No. (%)	
Better	61 (28.8)
Intermediate	81 (38.2)
Poor	70 (33.0)

Abbreviations: AML, acute myeloid leukemia; CEBPA, CCAAT/enhancer‐binding protein α; CK, complex karyotype; FAB classification, French‐American‐Britain classification; FLT3‐ITD, internal tandem duplications in the FMS‐like tyrosine kinase 3; IQR, interquartile range; MK, monosomal karyotype; NK, normal karyotype; NPM1, nucleophosmin 1; SD, standard deviation; WBC, white blood cell.

### 3.2 Comparison of lncRNA MVIH expression between AML patients and controls

**Figure 1:**
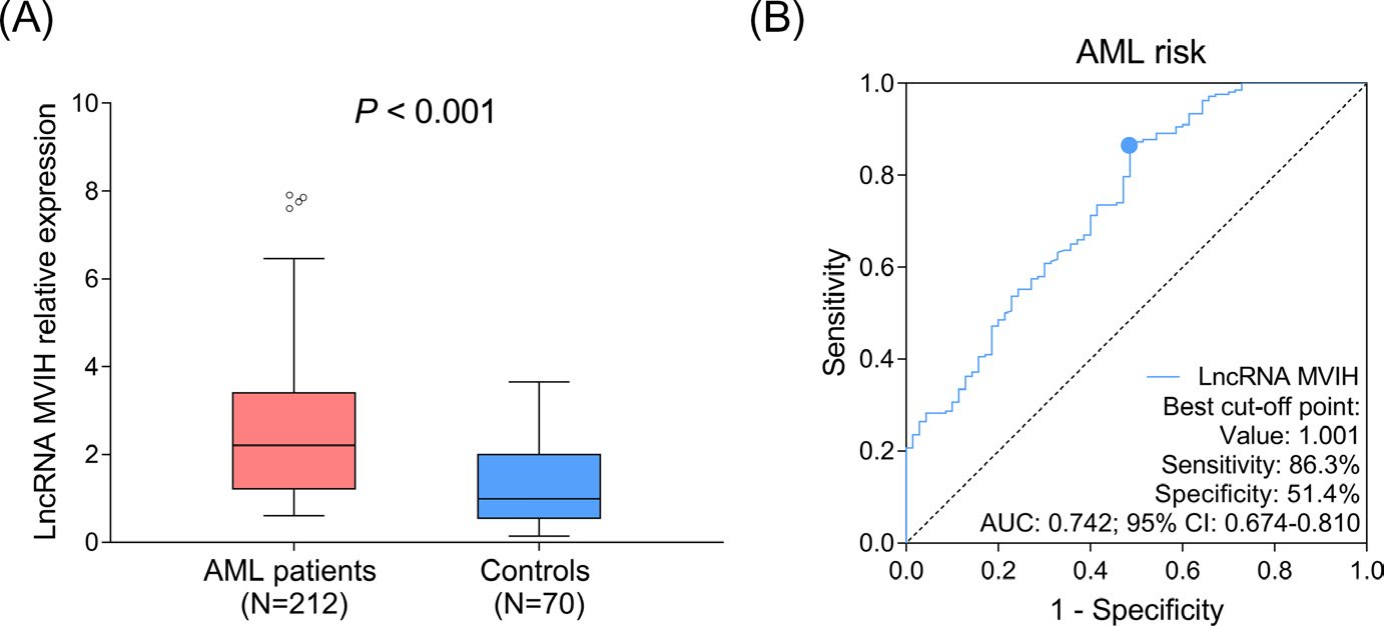
LncRNA MVIH expression in AML patients and controls. LncRNA MVIH expression in AML patients and controls (A). ROC curve for lncRNA MVIH predicting AML risk (B). The performance of lncRNA MVIH in differentiating AML patients from controls was assessed by ROC curves and the AUC. Comparison between two groups was determined by Wilcoxon rank sum test.* P* < .05 was considered significant. AML, acute myeloid leukemia; AUC, area under the curve; LncRNA MVIH, long non‐coding RNA microvascular invasion in hepatocellular carcinoma; ROC, receiver‐operating characteristic

LncRNA MVIH expression was increased in AML patients (median: 2.205 [1.221‐3.405]) compared to controls (median: 0.986 [0.544‐1.999]) (*P* < .001) (Figure 1A). Moreover, ROC curve showed that lncRNA MVIH had a good predictive value for elevated AML risk (AUC: 0.742 [95% CI: 0.674‐0.810]), meanwhile, lncRNA MVIH expression at the best cutoff value was 1.001, where AUC reached the maximum, and the sensitivity as well as specificity at best cutoff value were 86.3% and 51.4%, respectively (Figure 1B).

### 3.3 Correlation of lncRNA MVIH expression with FAB classification and risk stratification in AML patients

**Figure 2:**
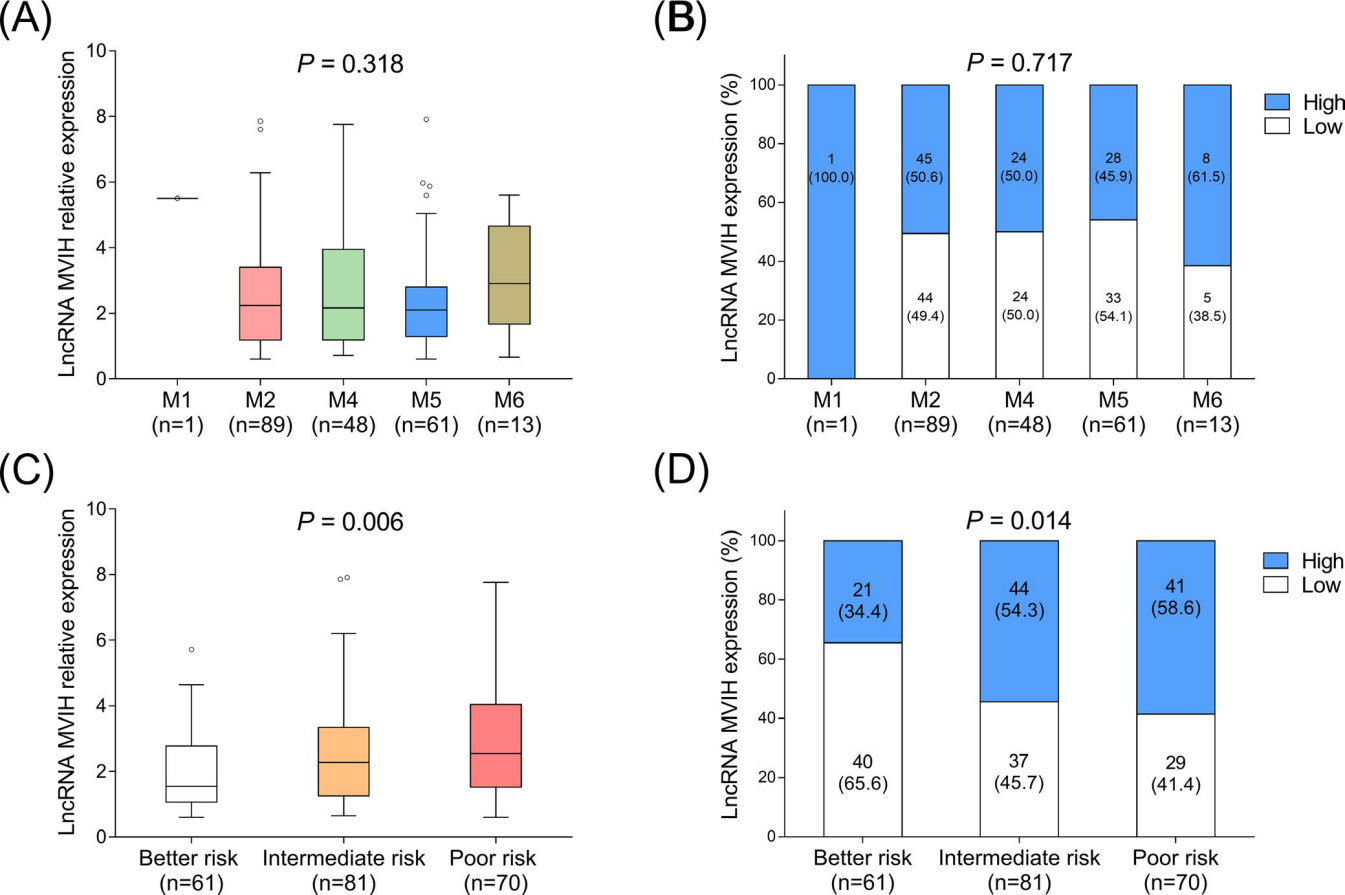
LncRNA MVIH expression in patients with different FAB classification and different risk stratification. LncRNA MVIH expression in FAB classification M1, M2, M4, M5, and M6 patients (A). Association of lncRNA MVIH high expression with risk stratification (B). LncRNA MVIH expression in better risk, intermediate risk and poor risk patients (C). Association of lncRNA MVIH high expression with risk stratification (D). Comparison of lncRNA MVIH expression among M1, M2, M4, M5, and M6 or among better risk, intermediate risk, and poor risk was determined by Kruskal‐Wallis H rank sum test. *P* < .05 was considered significant. FAB, French‐American‐British; LncRNA MVIH, long non‐coding RNA microvascular invasion in hepatocellular carcinoma

Regarding AML patients with different FAB classification, no difference of lncRNA MVIH expression was found among M1, M2, M4, M5, and M6 patients (Figure 2A,B). As to AML patients with different risk stratification, lncRNA MVIH expression was increased along with the worse risk stratification (Figure 2C,D).

### 3.4 Correlation of lncRNA MVIH expression with CR achievement and survival profiles in AML patients

**Figure 3:**
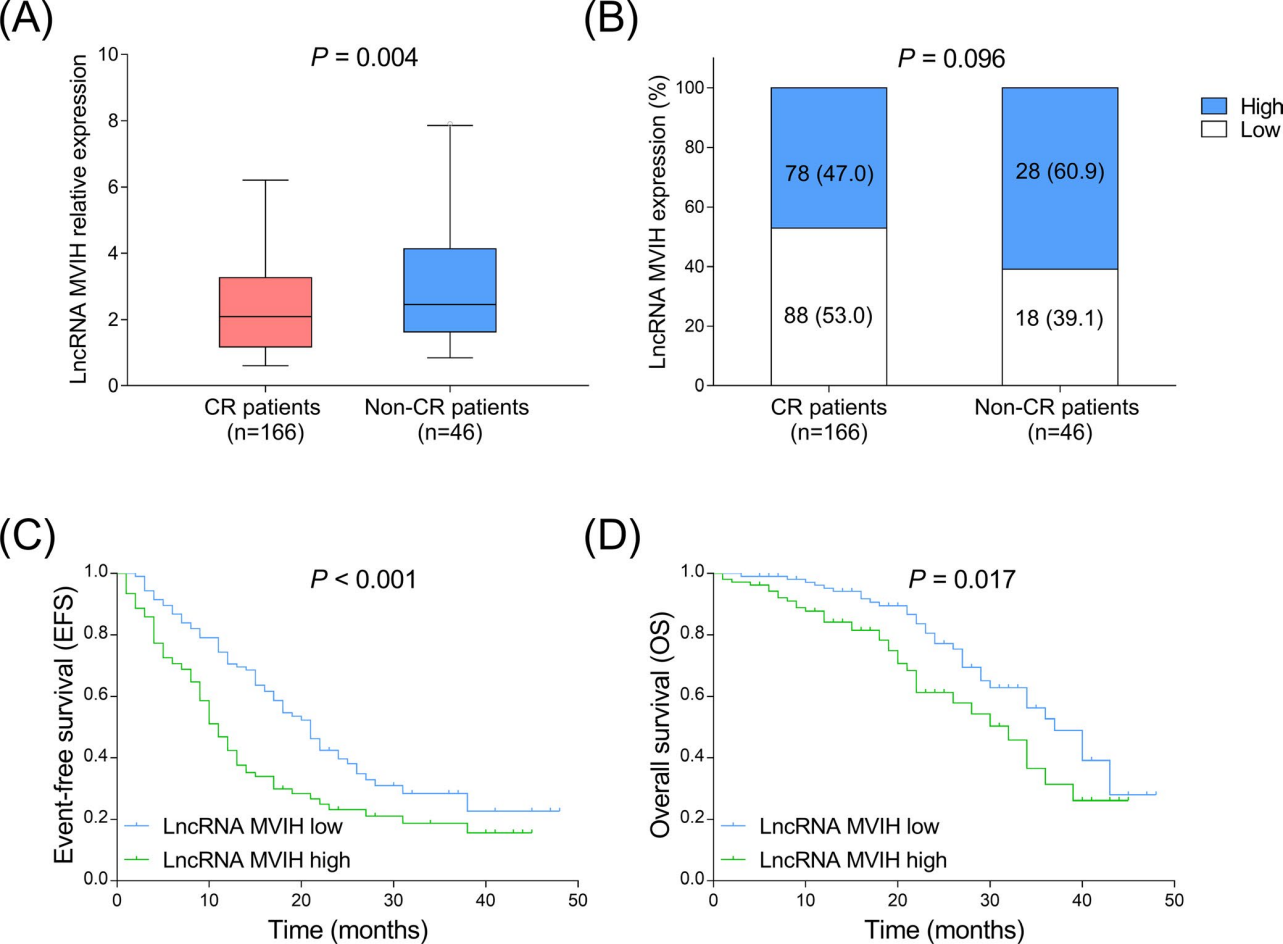
LncRNA MVIH expression negatively correlated with CR achievement, EFS and OS. Comparison of lncRNA MVIH expression between CR patients and non‐CR patients (A). Association of lncRNA MVIH high expression with CR achievement (B). Comparison of EFS between lncRNA MVIH high‐expression patients and lncRNA MVIH low‐expression patients (C). Comparison of OS between lncRNA MVIH high‐expression patients and lncRNA MVIH low‐expression patients (D). K‐M curves were used to exhibit EFS and OS. Comparison of lncRNA MVIH relative expression between CR patients and non‐CR patients was determined by Wilcoxon rank sum test. Comparisons of lncRNA MVIH high‐expression and low‐expression percentages between CR patients and non‐CR patients or among M1, M2, M4, M5, and M6 or among better risk, intermediate risk, and poor risk were determined by chi‐squared test. Comparisons of EFS and OS between two groups were determined by log‐rank test. *P* < .05 was determined by *t* test. CR, complete remission; EFS, event‐free survival; LncRNA MVIH, long non‐coding RNA microvascular invasion in hepatocellular carcinoma; K‐M curves, Kaplan‐Meier curves; OS, overall survival

In AML patients, 166 (78.3%) patients achieved CR and 46 (21.7%) patients did not achieve CR. LncRNA MVIH expression was decreased in CR patients compared to non‐CR patients (Figure 3A,B). Furthermore, both EFS (*P* < .001) (Figure 3C) and OS (*P* = .017) (Figure 3D) were shorter in lncRNA MVIH high‐expression patients compared to lncRNA MVIH low expression patients.

### 3.5 Effect of lncRNA MVIH on AML cell activities

**Figure 4:**
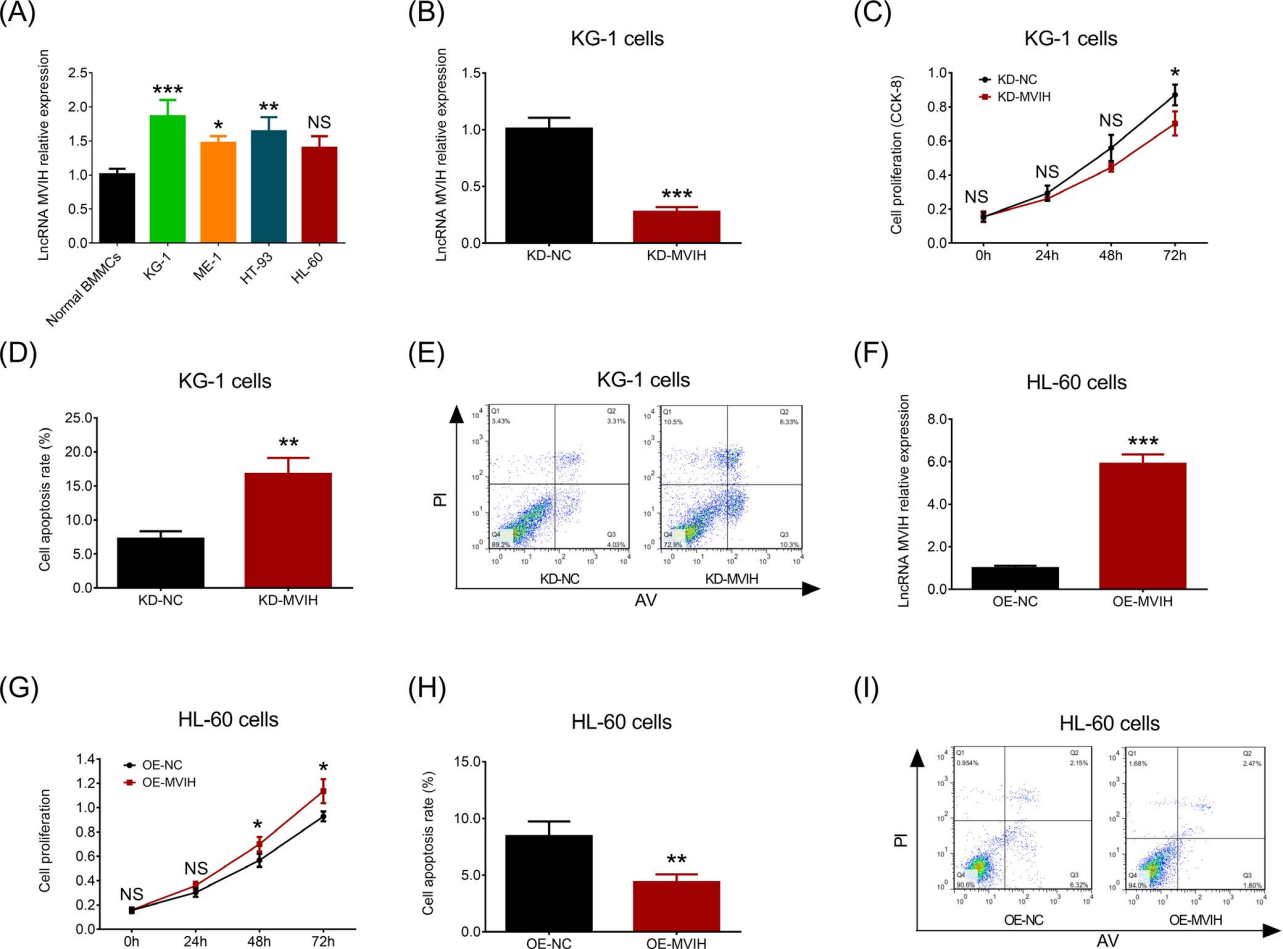
In vitro experiments. LncRNA MVIH expression in AML cell lines and normal BMMCs (A). LncRNA MVIH expression in KD‐MVIH group and KD‐NC group in KG‐1 cells (B). Cell proliferation in KD‐MVIH group and KD‐NC group in KG‐1 cells (C). Cell apoptosis in KD‐MVIH group and KD‐NC group in KG‐1 cells (D, E). LncRNA MVIH expression in OE‐MVIH group and OE‐NC group in HL‐60 cells (F). Cell proliferation in OE‐MVIH group and OE‐NC group in HL‐60 cells (G). Cell apoptosis in OE‐MVIH group and OE‐NC group in HL‐60 cells (H, I). Comparison of lncRNA MVIH relative expression among different cell lines was determined by Dunnett's *t* test. Comparison of lncRNA MVIH relative expression between KD‐NC and KD‐MVIH or between OE‐NC and OE‐MVIH was determined by unpaired *t* test. Comparisons of cell proliferation and apoptosis between KD‐NC and KD‐MVIH or between OE‐NC and OE‐MVIH were determined by unpaired *t* test. *P* < .05 was considered significant. LncRNA MVIH, long non‐coding RNA microvascular invasion in hepatocellular carcinoma; AML, acute myeloid leukemia; BMMCs, bone marrow mononuclear cells; KD, knock‐down; NC, negative control; OE, overexpression. ***P < .001; **P < .01; *P < .05

To explore the influence of lncRNA MVIH on cell activities in AML, we conducted some in vitro experiments. RT‐qPCR assay showed that lncRNA MVIH expression was increased in KG‐1 (*P* < .001), ME‐1 (*P* < .05), and HT‐93 cells (*P* < .01) compared to normal BMMCs, while no difference of lncRNA MVIH expression was found between HL‐60 cells and normal BMMCs (*P* > .05) (Figure 4A). Since the numerically highest lncRNA MVIH expression was observed in KG‐1 cells, we transfected lncRNA MVIH KD plasmids into KG‐1 cells, and lncRNA MVIH expression was dramatically decreased in KD‐MVIH group compared to KD‐NC group (*P* < .001) (Figure 4B). For cell proliferation, it was reduced in KD‐MVIH group compared to KD‐NC group at 72 hours (*P* < .05) (Figure 4C). For cell apoptosis, its rate was elevated in KD‐MVIH group compared to KD‐NC group (*P* < .01) (Figure 4D,4). Furthermore, the numerically lowest lncRNA MVIH expression was observed in HL‐60 cells, thus we transfected lncRNA MVIH OE plasmids into this cell line, and lncRNA MVIH expression was raised in OE‐MVIH group compared to OE‐NE group (*P* < .001) (Figure 4F). For cell proliferation, it was increased in OE‐MVIH group compared to OE‐NC group at 48 hours (*P* < .05) and 72 hours (*P* < .05) (Figure 4G). For cell apoptosis, its rate was reduced in OE‐MVIH group compared to OE‐NC group (*P* < .01) (Figure 4H,I). These data indicated that lncRNA MVIH was overexpressed in AML cell lines, and it promoted cell proliferation but inhibited cell apoptosis in AML.

## 4 DISCUSSION

LncRNAs are able to regulate gene expressions at various levels, such as chromatin modification, transcription, and post‐transcriptional processing.^8^ Increasing studies have revealed that lncRNAs play crucial regulatory roles in various pathological processes, especially in tumorigenesis.^13^ Remarkably, lncRNA MVIH, a newly found lncRNA that is initially reported to be upregulated in HCC, has been further explored and reported to be involved in the pathology of several cancers.^9^, ^12^, ^13^, ^14^ For example, lncRNA MVIH enhances cell proliferation and invasion through regulating matrix metalloproteinase (MMP) 2 and MMP9 protein expressions in NSCLC cells.^12^ Also, lncRNA MVIH facilitates cell proliferation and suppresses cell apoptosis via inhibiting miR‐199a in HCC cells.^13^ Besides, lncRNA MVIH activates tumor‐inducing angiogenesis via suppressing the secretion of phosphoglycerate kinase 1 (PGK1) in HCC cells.^14^ These data reveal that lncRNA MVIH may regulate some genes or enzymes such as miR‐199a, MMP9, and PGK1 to contribute to the initiation and progression of several solid tumors.

Other than the explorations of lncRNA MVIH in the pathology of solid tumors, a few clinical practices also disclose the lncRNA MVIH expression in some tumor tissues and the correlation of lncRNA MVIH expression with disease conditions in patients with solid tumors.^10^, ^11^, ^12^, ^13^, ^14^ For instance, some studies display that lncRNA MVIH expression is elevated in HCC tissues as well as breast cancer tissues than that in adjacent non‐cancerous tissues.^11^, ^14^ Additionally, a study discloses that lncRNA MVIH expression is positively correlated with Karnofsky performance score and World Health Organization (WHO) grade in glioma patients.^10^ Also, two other studies show that lncRNA MVIH expression is positively correlated with TNM stage in NSCLC patients as well as HCC patients.^12^, ^14^ These evidences reveal that lncRNA MVIH is overexpressed and correlates with worse disease condition in patients with several solid tumors. With this regard, we speculated that lncRNA MVIH might also be involved in the occurrence and development of hematological malignancies, including AML, whereas the exact role of lncRNA MVIH in AML or any hematological malignancies remains unknown. Hence, our study enrolled 202 AML patients to investigate the association of lncRNA MVIH expression with disease risk and some vital characteristics in AML, including FAB classification and risk stratification. We observed that lncRNA MVIH was overexpressed in AML patients, and it showed a good predictive value for increased AML risk. Additionally, although no correlation of lncRNA MVIH expression with FAB classification was observed, lncRNA MVIH high expression was found to be correlated with worse risk stratification in AML patients. These results might be due to: (a) overexpressed lncRNA MVIH indicated the excess ability of cell proliferation, and AML was characterized by the abnormal proliferation of undifferentiated and non‐functional leukemic blasts, indicating that lncRNA MVIH high expression might enhance the proliferation potential to facilitate the initiation of AML, thus lncRNA MVIH predicted increased AML risk^12^, ^13^; (b) lncRNA MVIH might regulate some genes that result in abnormal karyotypes and molecular mutations, thus its high expression correlated with worse risk stratification, while the detailed mechanism remained unclear.

The predictive role of lncRNA MVIH for prognosis in solid tumors has also been disclosed in some clinical practices.^10^, ^11^, ^12^ For instance, lncRNA MVIH high expression is associated with poor disease‐free survival and OS in breast cancer patients.^11^ Also, lncRNA MVIH high expression is associated with reduced OS in glioma patients as well as NSCLC patients.^10^, ^12^ However, little is known about the predictive value of lncRNA MVIH for prognosis in AML. In our study, we found that lncRNA MVIH expression negatively correlated with CR achievement as well as survival profiles including EFS and OS in AML patients. The possible reasons might be as follows: (a) lncRNA MVIH might induce resistance to chemotherapy in AML patients, thereby impeded the treatment efficacy and reduced possibility of CR achievement; (b) lncRNA MVIH might promote AML cell proliferation but suppress cell apoptosis through regulating some enzymes such as MMP2 and MMP9, which further accelerated disease progression and reduced survival time, thus lncRNA MVIH negatively correlated with EFS and OS in AML patients.^12^, ^13^, ^14^ Our data might provide support for further explorations about the predictive role of lncRNA MVIH for prognosis in AML, while some limitations still existed in our study: (a) although sample size (N = 212) was not small, further study with larger sample size was needed to validate our results; (b) follow‐up duration (median: 19.0 months) was relatively short, and long‐term assessment about the correlation of lncRNA MVIH with AML prognosis needed to be further explored.

To explore how lncRNA MVIH regulate cell activities in cancers, some in vitro or in vivo experiments are conducted by previous studies.^10^, ^11^ For example, lncRNA MVIH upregulation enhances cell proliferation, migration, and invasion but inhibits cell apoptosis in glioma cells.^10^ Another study displays that upregulated lncRNA MVIH promotes cell proliferation but inhibits cell apoptosis in breast cancer cells.^11^ However, evidence about the regulatory role of lncRNA MVIH in AML cells is limited. In our study, we found that lncRNA MVIH expression was elevated in several AML cell lines compared to normal BMMCs. Moreover, lncRNA MVIH promoted cell proliferation but reduced cell apoptosis in AML cell lines. These results might be on account of that lncRNA MVIH regulated some genes such as jun‐B and miR‐199a, which have been implicated as crucial regulators in the pathology of AML, and eventually enhanced AML cell proliferation but inhibit apoptosis.^16^, ^17^ Most importantly, our data implied that lncRNA MVIH might act as a possible treatment target in AML.

In conclusion, lncRNA MVIH may not only serve as a prognostic marker but also act as a therapeutic target in AML.
